# The Impact of Dietary Sphingolipids on Intestinal Microbiota and Gastrointestinal Immune Homeostasis

**DOI:** 10.3389/fimmu.2021.635704

**Published:** 2021-05-14

**Authors:** Johanna Rohrhofer, Benjamin Zwirzitz, Evelyne Selberherr, Eva Untersmayr

**Affiliations:** ^1^ Gastrointestinal Immunology Group, Institute of Pathophysiology and Allergy Research, Center for Pathophysiology, Infectiology and Immunology, Medical University of Vienna, Vienna, Austria; ^2^ Unit of Food Microbiology, Institute of Food Safety, Food Technology and Veterinary Public Health, University of Veterinary Medicine, Vienna, Austria

**Keywords:** sphingolipids, nutrition, immune modulation, gastrointestinal barrier, gastrointestinal microbiota, immunonutrition

## Abstract

The large surfaces of gastrointestinal (GI) organs are well adapted to their diverse tasks of selective nutritional uptake and defense against the external environment. To maintain a functional balance, a vast number of immune cells is located within the mucosa. A strictly regulated immune response is required to impede constant inflammation and to maintain barrier function. An increasing prevalence of GI diseases has been reported in Western societies over the past decades. This surge in GI disorders has been linked to dietary changes followed by an imbalance of the gut microbiome, leading to a chronic, low grade inflammation of the gut epithelium. To counteract the increasing health care costs associated with diseases, it is paramount to understand the mechanisms driving immuno-nutrition, the associations between nutritional compounds, the commensal gut microbiota, and the host immune response. Dietary compounds such as lipids, play a central role in GI barrier function. Bioactive sphingolipids (SLs), e.g. sphingomyelin (SM), sphingosine (Sph), ceramide (Cer), sphingosine-1- phosphate (S1P) and ceramide-1-phosphate (C1P) may derive from dietary SLs ingested through the diet. They are not only integral components of cell membranes, they additionally modulate cell trafficking and are precursors for mediators and second messenger molecules. By regulating intracellular calcium levels, cell motility, cell proliferation and apoptosis, SL metabolites have been described to influence GI immune homeostasis positively and detrimentally. Furthermore, dietary SLs are suggested to induce a shift in the gut microbiota. Modes of action range from competing with the commensal bacteria for intestinal cell attachment to prevention from pathogen invasion by regulating innate and immediate defense mechanisms. SL metabolites can also be produced by gut microorganisms, directly impacting host metabolic pathways. This review aims to summarize recent findings on SL signaling and functional variations of dietary SLs. We highlight novel insights in SL homeostasis and SL impact on GI barrier function, which is directly linked to changes of the intestinal microbiota. Knowledge gaps in current literature will be discussed to address questions relevant for understanding the pivotal role of dietary SLs on chronic, low grade inflammation and to define a balanced and healthy diet for disease prevention and treatment.

## Introduction

GI diseases are common in Western societies and associated with an increase in prevalence (1, 2). Estimations suggest 11% of the United States population (3) and 5-15% of the European population (with variations between Western Europe and Eastern Europe) (4, 5) are suffering from digestive diseases. This includes cancerogenic diseases affecting esophagus, stomach, intestines and pancreas, as well as inflammatory diseases like inflammatory bowel disease (IBD), esophagitis, coeliac disease, diverticular disease, alcoholic liver disease, acute and chronic pancreatitis and many more (5). Moreover, digestive disorders have been associated with psychosomatic manifestations, such as fatigue/neurasthenia, anxiety, phobia and panic disorders and pain syndromes (2). Although, current literature suggests to combine different scientific disciplines to identify functional relationships between immune, inflammatory and neurological disorders, analysis approaches involving data sets of the different scientific disciplines are limited. The increased prevalence of GI diseases is a major challenge for our health care systemassociated with increasing costs.

Recent demographic data suggest Western lifestyle to play a causative role in disease prevalence. In search for explanations of this phenomenon, investigators are increasingly focusing on the gut and its immune system. It is suggested that low grade and chronic inflammation rather than an acute defense reaction, might be the cause for disease development by slowly altering the immune response (6, 7). An inflammation causing a systemic response in the whole body would have effects on multiple organ systems. Besides manipulating the environment of affected tissue types towards tumorigenesis, a long lasting, low grade inflammation can also result in common chronic conditions, such as allergic diseases, autoimmunity, arteriosclerosis, obesity, insulin resistance and depressive disorders. By focusing on the gut as source of inflammation it is paramount to understand the role of nutrition on the cross-talk between the microbiome, GI barrier function and the gut associated lymphoid tissues (GALT). Western high fat diets have been reported to enhance intestinal inflammation by promoting gut permeability and altering gut microbiota (8, 9). In response, dietary modulation reduces severity of GI symptoms related to cancer, allergy and autoimmunity and has been suggested as a simple and commonly available approach to counteract disease onset and progression (10–12). Nutritional lipids have received much attention in the field of immuno-nutrition. Lipids are not only considered as energy storage molecules but have also been reported to be involved in the regulation of cell migration, the production of hormones and to act as second messenger molecules. These characteristics enable lipids to modulate immuneresponses (13, 14).

SLs are a highly diverse lipid class found in cellular membranes, lipoproteins and other lipid-rich structures, such as the skin. Their metabolites influence apoptosis, cell growth and cell migration. SLs contribute to pro- and anti-inflammatory immune responses. These lipid molecules are produced endogenously and their metabolism is strictly regulated. SLs are also found in food products, ingested and absorbed in the GI tract affecting its immune activation status and subsequently inflammatory and inflammation-related diseases. Previously reported mechanisms of SL action are inhibition of intestinal lipid uptake (15, 16), activation of pro- and anti-inflammatory receptors (17), lymphocyte chemotaxis (18), neutralization of bacterial endotoxins (19) and alterations of the intestinal microbiota (20–22). In context with the demographic changes of the last decades, the increasing prevalence of “civilization diseases” in Western societies and diet specific differences in SL content and composition, we suggest that dietary SLs contribute to the regulation of inflammatory stimuli.

Therefore, elucidating intestinal SL pathways and effector metabolism is crucial for identifying novel key players in immuno-nutrition to combat a dysregulated GI immune response.

## A Brief Introduction to SLs

Johann Ludwig Wilhelm Thudichum was the first who described SLs after identifying them as constituents in brain tissue. Due to their highly enigmatic nature, he named them after the ancient sphinx (23). The large and complex metabolism, the enormous amount of SL species and the lipophilic character of SLs have been huge obstacles for scientists. With the progress in genetic, molecular and technical methods during the past decades, a more lipid and enzyme centered approach was possible for investigations. Analysis of their detailed molecular structure and SL content of cells, tissues and food products is now possible.

### SL Structures

**Figure 1:**
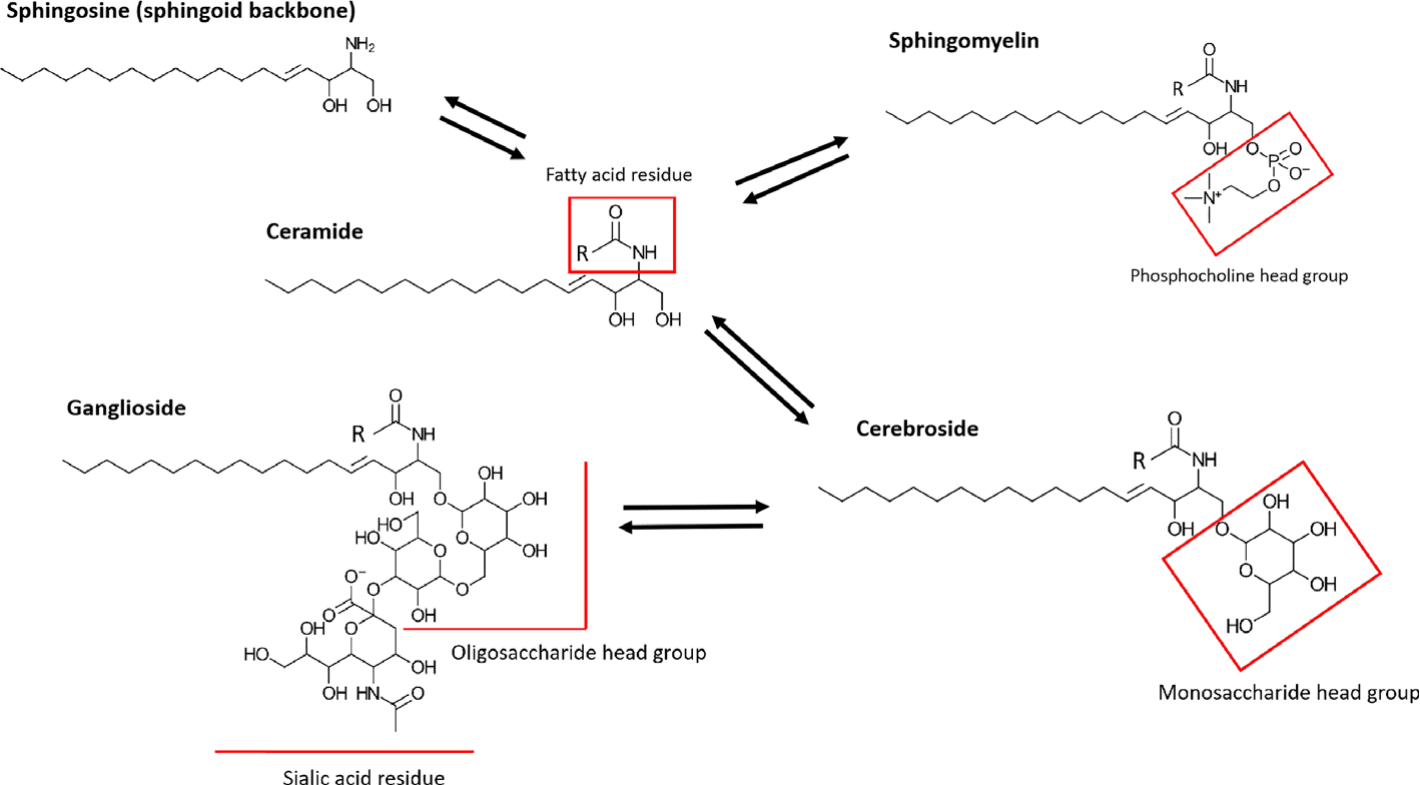
Simplified structures of prominent human SLs. SLs consist of a sphingoid backbone, which is represented by Sph in this figure. By linking a fatty acid residue *via* an amide linkage, Cer (N-acylsphingosine) is formed. Cers differ depending on length and saturation of their alkyl chain on the fatty acid residue. The further addition of a head group, such as phosphocholine or phosphoethanolamine, to a Cer results in the formation of SM. More complex glycolsphingolipids are generated by addition of carbohydrate head groups. Neutral cerebrosides are formed by linkage with monosaccharide head groups. Linkage to oligosaccharide headgroups with one or more sialic acid residues forms gangliosides.

SLs are a structurally highly diverse lipid class with over 4000 distinct SL subtypes (24). While most common in eukaryotic organisms, they are rarely found in prokaryotes (e.g. *Bacteroidetes* and *Proteobacteria*), archaea and viruses (25). Originally, SLs were described as components of cell membranes forming, together with cholesterol, phospholipids and proteins, membrane microdomains called lipid rafts, which are important for cell signaling pathways (26). SL metabolites emerged in studying inflammation, since their bioactivity was reported to regulate cellular signals involved in apoptosis and cell viability. SLs consist of a fatty acid linked to the amino group of a sphingoid base and a headgroup associated with a hydroxyl head group of the base (**Figure 1**). The sphingoid base sphingosine (Sph) is the most common SL backbone in mammals. However, different organisms display more than one single sphingoid backbone type. Human SLs, for example, have mostly Sph as backbone, but also sphinganine, 4- hydroxysphinganine, as well as small amounts of longer chain length homologs (27). Plant sphingoid backbones often display more double bonds along the alkyl chain than mammalian SLs. Cers are formed when a Sph backbone is linked to a fatty acid, typically with a length of 16 – 26 carbon atoms, without a head group. Usually, the term “Cer” refers to N-acylsphingosines. However, some studies do not distinguish Cers by their sphingoid backbones and all N-acyl-sphingoid bases are called Cers (27). By adding different head groups, such as phosphocholine, sugars or more complex carbohydrates, the SL types gain different identities and functions (28). The linkage to a head group, which consists of a phosphate group esterified to an alcohol, like phosphocholine or phosphoethanolamine, to Cers results in synthesis of the phosphosphingolipid sphingomyelin (SM). Glycosphingolipids are formed by linking the sphingoid backbone to a carbohydrate head group. This subfamily consists of the most diverse SL types and based on their carbohydrate compositions they can be either neutral or acidic. By associating with a monosaccharide like glucose or galactose, neutral cerebrosides are formed. Linkage to more than one saccharide forms neutral globosides. Binding of oligosaccharides, N-acetylglucosamine, N-acetylgalactosamine and one or more sialic acid residues on the sugar chain forms gangliosides.

### SL Metabolism and Catabolism

Although mammalian SLs consist of various species, their synthetic and catabolic pathways are shared. *De novo* synthesis of Cer starts with the condensation of a serine and palmitoyl-CoA to 3- ketosphinganine. The reaction is carried out by an enzyme called serine palmitoyltransferase. 3- ketosphinganine is then further reduced to sphinganine and acylated to dihydroceramide by one of six mammalian Cer synthases. Each of the Cer synthases has its own preferred acetyl-CoA substrate.

By introduction of a double bond at the sphingoid base a Cer is formed, which represents the branch point of SL metabolism. Cer synthesis takes place at the cytosolic leaflet of the endoplasmic reticulum (ER) (24). For further and more complex metabolism, Cers have to be transported to the Golgi apparatus. Here different head groups are added to the Cers. This results in formation of SM by the enzyme SM synthase, or to glycosphingolipids, such as cerebrosides and gangliosides, by respective enzymes. Additionally, Cer can also be produced by turnover of these more complex SLs i.e. *via* hydrolysis of SM and glycosphingolipids. Lastly, Cer is formed by recycling of SL metabolites, such as S1P or C1P, in the salvage pathway.

The most intensely studied catabolic pathway starts with the hydrolysis of SM to phosphocholine and Cer by alkaline, neutral and acid sphingomyelinases (SMases) (29, 30). Alkaline SMase is most abundant in the plasma membrane and endosomes of the microvilli enterocytes and works optimally at pH 8.5 – 9. Noteworthy, differing from neutral and acid SMases, the alkaline SMase is restricted to the intestinal mucosa and to the liver in humans, where its activity is much higher than those of the other SMases. As an ectoenzyme, alkaline SMase is located on the cell surface and can be released into the lumen by both bile salt and pancreatic trypsin. Neutral SMases work preferentially at pH 7.5 and are located at the ER, nucleus, Golgi apparatus, plasma membranes and mitochondria. Acid SMases function below pH 5.5 and are mainly found in lysosomes. It can also be secreted by mast cells in response to inflammatory stimuli (31). After the hydrolysis, Cers can be either converted into ceramide-1-phosphate (C1P) by Cer kinase or be further degraded by one of six mammalian ceramidases. As a result, Sph and fatty acids (FAs) are produced. Endogenous Sph production is restricted by the breakdown of Cers (24). Furthermore, SM and Cer cannot be absorbed by enterocytes, in contrast to Sph and its metabolite S1P. S1P is generated by the phosphorylation of Sph by Sph kinase (SphK)1 and 2 (18, 32) and has two possible metabolic fates. It can either be dephosphorylated back to Sph by S1P phosphatases or degraded irreversibly by S1P lyase to phosphoethanolamine and hexadecenal, which will be used for acyl-CoA synthesis.

### The Impact of Dietary SLs

**Figure 2:**
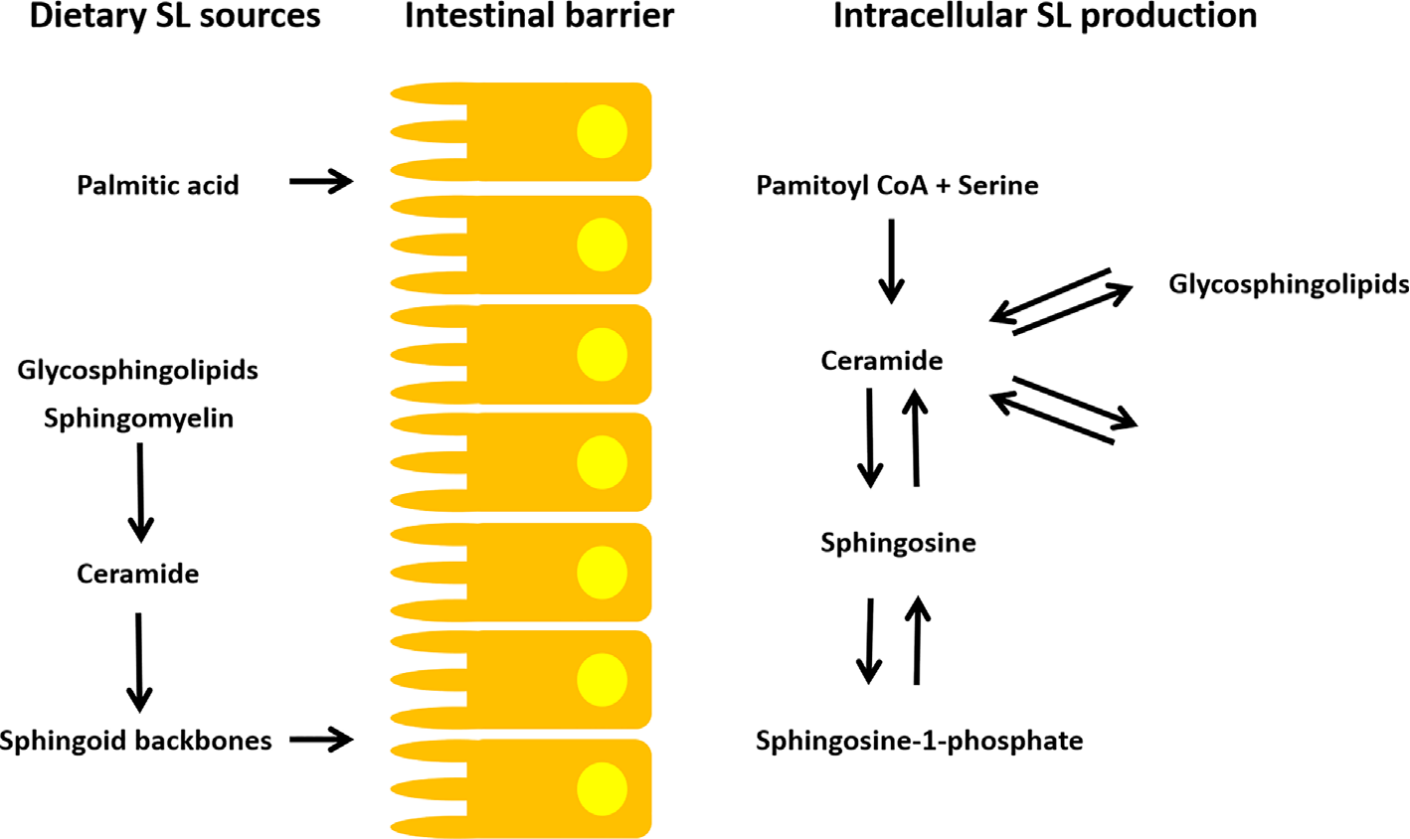
SL metabolism of dietary and intracellular SLs. A luminal breakdown to sphingoid backbones is required for intestinal absorption of exogenous SLs. Additionally, dietary palmitic acid consumption is suggested to modulate endogenous SL production (35, 36). The endogenous SL metabolism is highly complex. Cers are the branch point of SL homeostasis. They are synthesized *via* SM/cerebroside hydrolysis, *de novo* synthesis (palmitoyl-CoA + serine) and *via* the salvage pathway (e.g. Cer to S1P/C1P and vice versa). Subsequently, many SL metabolites can be generated from the Cer building blocks.

In the last decades investigations on the increasing prevalence of diseases common for developed societies have focused on the impact of westernized dietary habits. Nutritional patterns including the frequent intake of high levels of protein, sugar, fat, salt and cholesterol have been suggested to promote chronic GI inflammations affecting immune responsiveness and subsequent diseases in multiple organ systems. With only minor amounts of a few micromoles per kilograms in fruits to several millimoles per kilogram found in dairy products, eggs and soybeans, the SL content varies enormously among nutritional compounds (25). On average, adults on a Western diet consume 0.3-0.4 g SLs per day mainly derived from SM (33). In Asian diet a lower amount of milk SM and much more cerebrosides are ingested (34). In the Western population, the intake of plant SLs is estimated to be 50 mg/day, although it can be much higher for vegetarians. Dietary SLs require a luminal breakdown to sphingoid backbones before intestinal absorption (35, 36) and differ in their structure depending on their origin (plant or animal SLs) (**Figure 2**). Various food products have been analyzed regarding their overall SL content [a detailed list is provided by Vesper et al. (25)]. However, the exact SL composition of different food products and diet-specific differences are still not well examined. Enhanced accessibility to high performance liquid chromatography (HPLC), gas chromatography-mass spectroscopy (GC-MS) and matrix-assisted laser desorption/ionization-mass spectrometry (MALDI-MS) approaches enable analysis of SL structure and quantity in different food sources (**Table 1**). First results have been highly interesting as they provide a more detailed insight into SL homeostasis. The uptake of plant sphingoid bases has been recently demonstrated to be less efficient in the small intestine. A study in rats suggested an efflux mechanism which allows enterocytes to release plant sphingoid bases back into the lumen (44). Despite possible effects on the host SL metabolism, higher amounts of plant Sph, together with non-digested SM and non- degraded Cer, is released into the colon. Possible downstream effects on the microbial gut community remain to be elucidated. Most SLs of mammalian food products consist of various SL types, such as SMs, cerebrosides, gangliosides, globosides or sulfatides. They are linked to a broad spectrum of different head group components (45). Plant SLs mainly consist of cerebrosides and phosphoinositides with glucose, galactose, mannose and inositol (46). Although, dietary SLs are not required for survival, they influence the composition of the gut microbiome and affect subsequent immune responses in the GI tract. By exploring underlying mechanisms, SL homeostasis has been suggested as a universal stress response. Considering the ongoing demographic changes, the healthcare system will be confronted with a huge burden. In view of the Western diet as an initial trigger for disease onset, a change in diet is a cheap, commonly accessible and most powerful tool to combat overall morbidity and increasing health care costs. Identifying diet-specific differences in SL composition is of uttermost importance to elucidate the accompanying role of SL metabolism in health and disease.

**Table 1:** Summary of SL content in important food sources.

SL metabolite	Dietary Source	mg/100g	References
Sphingomyelin	Bovine milk (whole)	9	(38, 39)
	Cottage cheese	139	
	Buttermilk quark	74	
	Egg yolk	82	
	Beef	44-69	
	Mackerel	224	
	Human breast milk	3-14	
Cerebrosides (total)	Wheat flour	226	(40)
	Soybean	310	
	Barley	275	
	Rice	11.5	
	Corn	11.5	
	Pumpkin	145	
	Cabbage	36.5	
	Spinach	192.9	
	Broccoli	112	
	Sweet potato	67	
	Potato	15.6	
Glucosylceramides	Sugar beet*	12-45	(39, 41)
	Potato*	1-20	
	Apple*	49- 94	
	Wheat*	20	
	Soybean*	20-39	
	Bovine milk	0.7-1.9	
Lactosylceramides	Bovine milk	0.8-1.2	
Gangliosides	Bovine milk	0.5-11	(39, 42, 43)
	Anchovies	9.9	
	Egg yolk	16	
	Chicken (liver)	29	
	Chicken (meat)	0.4–1.5	
	Beef	0.3-0.9	
	Pork	0.5	
	Crab	0.5	
	Squid	0.7	
	Human breast milk	19 - 26 (mg/l)	

*Information was obtained from a study examining dry by-products of the food industry.

Information on quantitative content of different SLs is limited and biased by the central research question of the studies. Quantification of the most abundant SLs in dietary sources is pivotal to define nutritional recommendations suitable for balancing SL homeostasis. When evaluating differences in Western versus vegetarian or vegan diets, there is a urgent need of quantitative examining cerebroside composition in food. Content of SM has been reported to vary depending on diet and maintenance of the animals (37).

## SLs and Inflammation

SL metabolites are well known to play a pivotal role in inflammatory signaling pathways. Low grade inflammation initiated by a Western diet cannot be detected *via* commonly available clinical biomarkers. However, constant stress and activation stimuli are suggested to be powerful promotors of metabolic changes (24, 47). Besides the detrimental impact of Western diet on health, a well-balanced nutrition might have a preventive and therapeutic function.

### Sphingomyelin

SM is an important constituent of cell membranes. It is associated with cell signaling by the production of lipid-soluble second messenger molecules and by the formation of lipid rafts. It influences apoptosis through the degradation to Cers. SM has been discussed as a dietary modulator of cholesterol synthesis. By establishing a complex network of H-bonds, it interferes with cholesterol absorption slowing down its transfer to enterocytes. Via the same mechanism, it is suggested to interfere with triglycerides and FAs reducing serum and hepatic lipid concentrations in a dose dependent manner (48, 49). Investigations reported different inhibition patterns of intestinal cholesterol absorption when administering egg or milk SM. A stronger inhibitory effect of milk SM was suggested to cause a higher degree of saturation of fatty acyl groups (15, 16). An investigation on milk SM deriving from milk fat of Holstein cows and Jersey cows identified differences in SM content, which may be induced by breed, diet and stage of lactation (37). Similar results were found for analyses of soybean SLs. Significant differences in overall glucosylceramide (83.4−397.6 nmol/g) and major Cer (8.4−20.7 nmol/g) contents have been detected by comparison of 15 soy bean lines (50). Variations between SMs from different food products, as well as batch-to-batch variations, are important to consider when interpreting study results (37).

### Cer and C1P

Cer is a pro-apoptotic molecule. It has been reported to work *via* caspase-dependent and independent mechanisms (24). Additionally, cellular Cer content has been linked to inflammation and metabolic diseases. Cers are synthesized *via* three endogenous pathways (SM/Cerebroside hydrolysis, *de novo* synthesis, salvage pathway). Due to several degradation mechanisms, the level of Cer metabolites is stable. By an increased generation of Cers or by prevention of degradation, an accumulation of Cers may occur provoking excessive apoptosis. Tumor necrosis factor (TNF)-α activates SMases leading to an accumulation of Cers. Elevated TNF-α levels are associated with lipotoxicity by activation of caspases, protein kinase C, serine/threonine protein phosphatase and cathepsin D activity (51). Moreover, promotion of insulin resistance is observed by antagonizing insulin signaling (52). HPLC, GC-MS and MALDI-MS approaches recently revealed insights into the complexity of dietary Cer structures, suggesting a much more complex function of the SL metabolite (53, 54). In accordance with this theory, recent studies reported not only pro- inflammatory effects of Cers. There is increasing evidence that higher Cer content of cells can prevent Lipopolysaccharide- (LPS-) stimulated inflammatory responses (24). Moreover, Cers produced in genetically modified yeast was reported to inhibit TNF-α signaling resulting in stable cell viability (55). Also, orally administered plant derived Cer-precursor SLs have been used as dietary supplements to restore skin barrier function in humans (56).

The Cer metabolite C1P has emerged as a bioactive SL metabolite involved in cell proliferation, macrophage migration, and inflammatory response. It prevents cell death in bone marrow derived macrophages and inhibits activation of caspases. It was further demonstrated to block alkaline SMase and subsequently the formation of Cers, suggesting C1P to antagonize Cer function (57). Mechanisms involving C1P have been described to be mainly located in intracellular compartments. Cer kinase is activated by different agonists, such as IL-1β, macrophage colony stimulating factor or calcium ions. Knock-down of the C1P transfer protein in mice resulted in increased levels of IL-1β and IL-8 levels and enhanced inflammasome assembly (58). A recent investigation has demonstrated a G (i) protein coupled plasma membrane receptor, suggesting C1P as an extracellular ligand to mediate chemotaxis (59). By stimulating phagocytosis in neutrophils and activating degranulation in mast cells, C1P is thought to respond to inflammation (60, 61). C1P reduces TNF-α production by inhibiting its post-translational modification (19, 62). In summary, these data suggest C1P involvement in inflammation as a feedback regulation to Cer stimuli. However, its precise role in inflammation remains to be elucidated.

### Sph and S1P

Sph are the most common sphingoid backbones of mammalian SLs. Their presence increases the permeability of phospholipid membranes. Moreover, Sph has been described to act like the structural counterpart of glycerol (63). Of interest, dietary D-erythro-Sph has been suggested to protect the human skin by altering skin microbiota (64). Topical Sph was suggested as effective treatment option for microbial skin diseases (65). It is still matter of discussion whether the skin barrier-improving effects only depend on the microbiota alteration or Cer synthesis activation in the skin. Current research further aims to evaluate Sph as topical and protective antibiotic (64, 65) and as anti-proliferative agent (63).

S1P derives from ingested SM or cellular membrane SM, which is converted into Sph and phosphorylated to S1P by Sphingosine Kinases (SphKs). SphKs 1 and 2 are highly expressed in lung, small intestine, spleen and stomach (66). Although, most cells are producers of endogenous S1P, the SL is subsequently degraded by S1P lyase, which is found in high levels in tissues such as the small intestine (67). In most tissues S1P levels remain at a baseline. In lymph and blood, S1P levels range from low micromolar to several hundred nanomolar due to the lack of S1P lyase or an enhanced SphK activity (68, 69). Platelet derived growth factor 6 induces Cer production by SM hydrolysis, which is then further metabolized to Sph and S1P. Previously, S1P has been reported to work intrinsically as well as extrinsically. It serves as a second messenger regulating calcium homeostasis in the cell. Its extracellular functions depend on five membrane-bound G-protein coupled receptors (S1P1-5). S1P1 has been reported to contribute to elevated vascular integrity by effecting endothelial adherence junctions. S1P2 and S1P3 are considered to improve vascular contraction. A lack of S1P2 is associated with vascular barrier leakage (70). Recent findings reported a protective effect of enhanced blood S1P levels in allergic mice, suffering from anaphylaxis, as well as a faster recovery after anaphylaxis by enhanced clearance of mast cell mediators (32). In contrast, enhanced tissue S1P levels have been reported to promote inflammation. S1P1-5 are expressed on the surface of several lymphocyte cell types including mast cells and eosinophils (71, 72) suggesting S1P to be crucial for immune cell migration and activation. Especially, S1P1 and S1P2 are important regulators of mast cell and eosinophil responses. Mast cells have been reported to be potent producers of endogenous S1P due to enhanced SphK activation (18). Alterations in S1P homeostasis by deletion of SphK1 or SphK2 were demonstrated to affect sensitization and effector phase in food allergy. Splenocyte analysis by flow cytometry found reduced populations of CD4+ effector T-cells in both SphK knock out strains, as well as a reduced allergy effector cell influx in the gastric mucosa. Enhanced barrier permeability was detected in CaCo2 monolayer stimulated apically with S1P (73). This provides evidence that presence of SphK influences allergen uptake by regulation of the GI barrier integrity. Therefore, S1P effects might depend on a concentration gradient. While enhanced blood S1P levels might be beneficial for barrier integrity, an increase in tissue S1P levels promotes barrier disruption and inflammation. Considering the dietary uptake of SLs, an enhanced need for S1P lyase activity in the small intestine seems logical. However, higher serum S1P levels together with Cer metabolites such as N,N-dimethylsphingosine have been reported to be associated with enhanced anxiety-like behavior in studies investigating associations between SL homeostasis and psychologic disorders and pain syndromes (74, 75). Without any doubt, additional investigations on the metabolite gradient need to be performed.

## SLs and the Gut With Focus on GI Barrier Integrity

**Figure 3:**
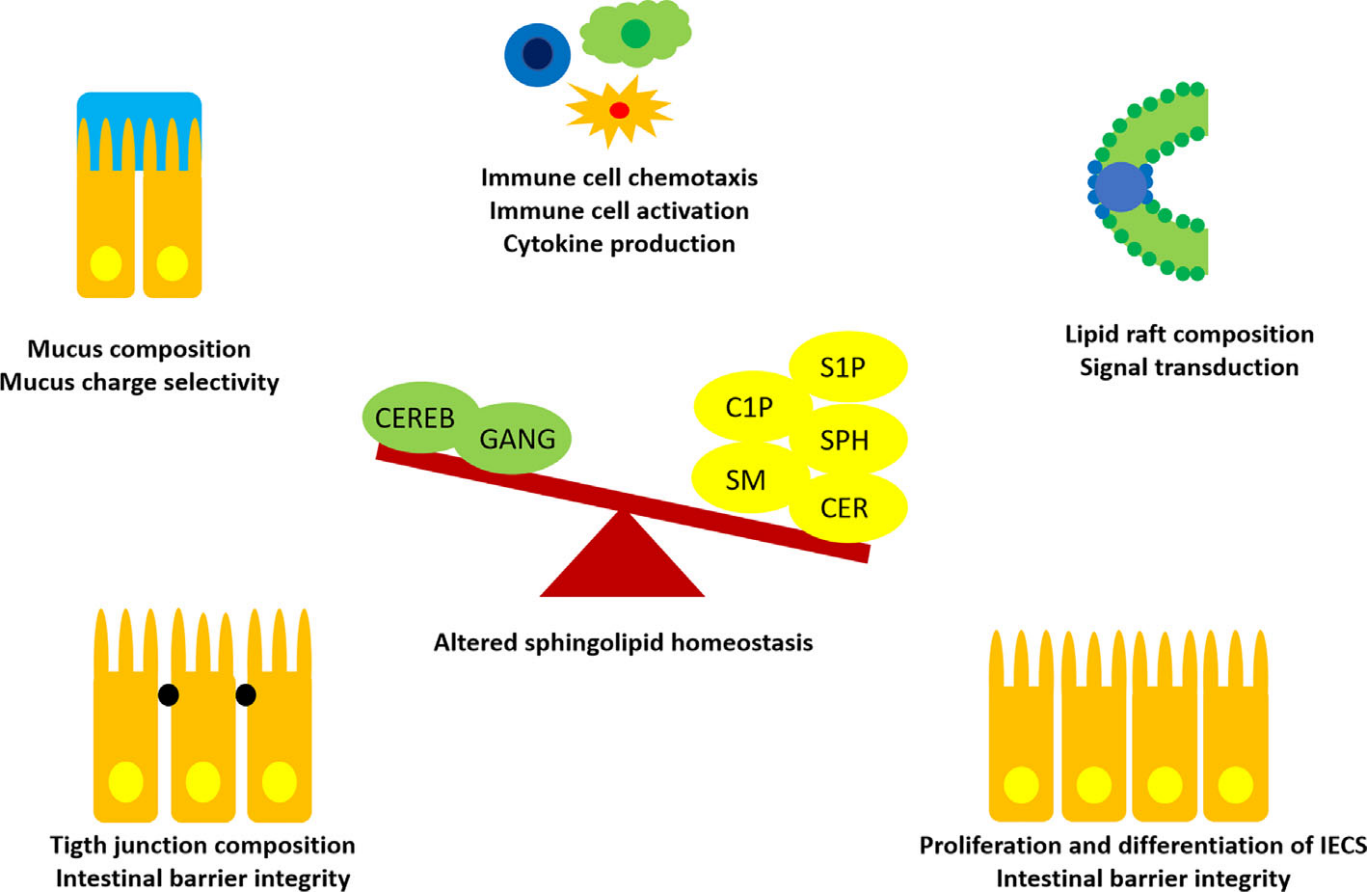
Influences of SLs in intestinal homeostasis. Recent findings suggested an imbalance of SLs and metabolites in favor of S1P, C1P, Cers (CER), Sph, SM, instead of more complex cerebrosides (CEREB) and gangliosides (GANG) to promote intestinal inflammation. SLs are able to interact with IECs and immune cells at different sites. Thus, SLs are promising candidates for treatment of immune-mediated diseases and as predictive biomarkers.

As essential structural components of GI cell membranes, SLs influence barrier integrity and function (**Figure 3**). The GI tract is lined by a single cell layer of different, constantly self-renewing epithelial cell types, termed intestinal epithelial cells (IECs). The luminal side of this single-cell barrier is covered with an alkaline, antimicrobial mucus layer, protecting the barrier from direct contact with the commensal gut microbiota and digested nutritional compounds. On the basolateral side, the GALT is located, which consists of lymphocytes being nourished by blood and lymph vessels and surrounded muscles embedded in loose connective tissue (8).

### SLs Influence Cell Differentiation Along the Crypt-Villus Axis

SLs are found throughout the whole GI tract, but preferably located in the apical membrane of IECs (67). Differences in SL distribution have been reported with a higher concentrations of Sph and glucosylceramide in the villi and trihexosylceramide in the crypts (76). This suggests that specific SL metabolite distributions along a crypt-villus axis may stimulate cell differentiation. In support, five of the six Cer synthases have been found in intestinal mucosal cells (33). Recent investigations suggest an imbalance in intestinal SL metabolite distribution to enhance inflammation. Enhanced levels of SM, Cer, S1P and C1P and decreased levels of cerebrosides and gangliosides were found in rodent studies of dextran sulfate sodium colitis (77). Moreover, high levels of SM and Cer have been detected in ilea of Crohn’s disease patients (78). TNF-α was demonstrated to upregulate the *de novo* synthesis of Cers in colon cancer. In contrast, inflamed intestinal tissue showed decreased levels of gangliosides, which was reversable by dietary ganglioside supplementation (79). Thus, dietary supplementation of specific SLs might act beneficially in gut inflammation by preserving morphological features important for intestinal function and immune homeostasis (33, 76, 80).

### Leaky or Tight? – Interaction of SLs With Cell Junction Related Proteins

It is well known that a disruption of the intestinal cell junctions enhances GI barrier permeability. As a consequence, luminal food and microbial antigens increasingly pass the GI barrier and might interact with GALT resulting in an altered immune response. This “leaky gut” is common in patients with digestive disorders. Absorption of LPS is increased, leading to chronic inflammation, which initiates the onset of metabolic diseases typical for Western societies, such as diabetes and non-alcoholic fatty liver disease (81, 82). Exogenous SMase was previously demonstrated to increase transepithelial permeability. At concentrations as low as 0.01 enzyme units/ml, transepithelial resistance was decreased. The barrier disruption was associated with accumulations of Cers and simultaneously decreased SM and cholesterol levels in membrane fractions containing tight junction proteins occludin and claudin-4 (83). Also, decreased amounts of cholesterol at the plasma membrane resulted in failure of occludin and claudins to localize at the tight junctions (84). A better understanding of the underlying mechanisms of tight junction formation is important to clarify the role of dietary SLs on intestinal barrier integrity.

### Combating Inflammation by Changes in Lipid Raft Composition

Lipid rafts are microdomains in plasma membranes rich in SLs and cholesterol, which harbor a variety of signaling and transport proteins. They are specialized in signaling allowing a closer interaction of protein receptors for signal transduction due to kinetically favorable conditions (85). In enterocytes, the brush border is a highly specialized membrane designed to absorb dietary nutrients and to simultaneously form a barrier towards luminal pathogens. Cholesterol or caveolin depletion in membranes was shown to inhibit inflammatory signaling by disrupting microdomain structure. Dietary ganglioside-induced reduction in cholesterol content reduced pro-inflammatory mediators in the intestinal mucosa after acute exposure to bacterial endotoxin (79). Moreover, a dysregulation in lipid rafts initiated by an accumulation of Cers was found in primary bronchial epithelial cells of cystic fibrosis patients (86). Cholesterol rich lipid rafts were reported to enhance inflammatory activity of TLR4 and 3 agonists. A depletion of cholesterol downregulated inflammatory signaling by TLR4 (87). Thus, effects of dietary SLs on lipid raft formations are promising targets for further studies in inflammatory diseases.

### SLs and Their Impact on Mast Cell Activation

Interactions between mast cells and modulating lipids are recently emerging and provide novel insights in underlying mechanism of the gut-brain axis. Mast cells are tissue resident immune cells, often located in skin and mucosa. They are well known for their secretory granules, which degranulate upon mast cell activation and release mediators such as histamine, proteases and cytokines. Mast cells are of high relevance in allergy research, since they respond to allergen exposure with IgE-mediated degranulation leading to tissue damage. Recently, their extensive, uncontrolled degranulation has been associated with an irritable bowel syndrome (IBS) and myalgic encephalomyelitis/chronic fatigue syndrome (ME/CFS), both strongly associated with infections or stress situations (88, 89). Mast cell interactions with cells of the central nervous system are considered to link stress with GI symptoms. Furthermore, degranulation of mast cells is calcium-dependent. The entry of calcium is modified by Cers, which can be produced from SM by acid SMase. A rodent study demonstrated exogenously stimulated Cer production to trigger apoptosis of mast cells in acid SMase knock-out mice (31). Thus, exogenously stimulated Cer production might be a powerful tool for dietary supplementation in situations of mast cell activation. Dietary recommendations focusing on mast cell activation have been suggested to combat symptom severity in allergy, IBD, IBS and ME/CFS (89). However, the exact role of dietary SLs in excessive mast cell activation and effects on the gut-brain axis has to be elucidated in further studies.

### SLs and the Mucus Barrier

Mucins are a family of heavily glycosylated proteins produced by specialized epithelial cells. They contribute to epithelial barrier function and prevent microbial invasion. In turn, certain members of the gut microbiota (commensals and pathogens) are also able to efficiently break down mucins (90). In line, bacteria competing with pathogens for the degradation of mucins are considered as gatekeepers for a healthy gut and have shown great potential as probiotics (91). Host membranes are protected from bacteria through the secretion of alkaline mucus. However, the effect of charge selectivity changes depending on mucus composition. Interactions with the headgroups of SM or phosphatidylcholine are suggested to alter intestinal mucus function. In accordance, intestinal mucus samples of ulcerative colitis patients demonstrated reduced phosphatidylcholine levels compared with healthy controls (92). Alkaline SMase was shown to inactivate a pro-inflammatory platelet activating factor, thus, being able to counteract intestinal inflammation (93). Therefore, the impact of bacterial, neutral and acid SMases have to be considered when studying SL metabolism in the gut. Data on bronchial mucosal barrier functions are available, due to intensive research on SLs in respiratory diseases. These results might assist investigations on GI mucosal barrier function. Bacterial SMases were found to strongly inhibit transmembrane conductance regulator function in cystic fibrosis reducing mucosal fluidity (94). Analyses of sputum revealed enhanced levels of SM and glycosphingolipids in samples of cystic fibrosis patients (95). A study examining the association of plasma S1P levels with CFTR function and clinical symptom presentation found reduced levels of unbound plasma S1P accompanied by GI symptoms in cystic fibrosis patients (96). Furthermore, enhanced Cer accumulation through neutral SMase activity was monitored in a study investigating the sputum of smokers. The enzyme is activated by TNF-α and interferon-gamma (IFN-gamma) stimuli (97). However, differences in signaling pathways activated by alkaline, neutral, acid or bacterial SMases still remain elusive.

## SLs and Microbiota

### Microbial SL Metabolism

While the importance of mammalian SL turnover for mediating various cellular processes is well recognized, mechanisms of microbial SL metabolism are not adequately understood. Prokaryotic SL production has first been described in members of the phylum *Bacteroidetes* (e.g. *Bacteroides, Prevotella, Porphyromonas, Sphingobacterium*) (98) and more recently also in some *Proteobacteria* (e.g. *Sphingomonas, Bdellovibrio, Acetobacter*) (99, 100). Of interest, many of the known bacterial SL producers are also associated with eukaryotic hosts, indicating a close symbiotic relationship. In fact, SL-mediated bacteria-host interactions have been unveiled in a number of plants, animals, as well as unicellular eukaryotes, suggesting an evolutionary early development of this trait (101). The potential influence of bacterial SL production on the host is further underpinned by the abundance of the *Bacteroidetes* phylum, constituting up to 30-40% of the human gut microbiome (101). The gut microbiome represents a highly complex ecosystem with a large potential to influence host health, which has been extensively studied in the last decade. However, the extent of impact of bacterial SLs on eukaryotic physiology, metabolic processes and immune homeostasis is not fully understood yet.

The majority of bacterial SLs are still not characterized and recent studies just begun to uncover the diversity of bacterial SL structures. For example, *Alistipes* and *Odoribacter* species have been found to be responsible for sulfonolipid production in mouse cecum, which correlated with a high-fat diet (102). Additionally, *Bacteroides fragilis* has been shown to produce three different types of SLs, namely, the Cer phosphorylethanolamine, its corresponding dihydroceramide base, and the glycosphingolipid α- galactosylceramide (103). These and other bacterial-derived SLs can pass the epithelial barrier in the gut and enter host metabolic pathways, [as shown by Johnson et al., 2020 (104)] They administered a SL-producing *Bacteroides thetaiotamicron* to mice and observed increased levels of Cers in liver and reduced *de novo* SL production, which was not achieved when the *B. thetaiotamicron* capability to produce SLs was knocked out (104). In another study mono-colonization of germ-free mice with a SL-deficient *B. thetaiotaomicron* strain led to intestinal inflammation and shifts in SL levels in the intestine (105). Moreover, IBD patients have decreased *Bacteroides*-derived SLs but increased host SLs, further highlighting the role of bacteria-derived SLs in intestinal immune homeostasis (106). Likewise, Duan et al. showed that germ-free mice have a reduced SM hydrolysis capability, suggesting that the intestinal microbiota contributes to SL turnover (107). Thus, microbial SLs have the potential to mediate signaling pathways and influence their hosts lipid metabolism (108). However, the exact mechanisms underlying SL- mediated host-microbial interactions and their implication in diseases warrant further research.

Diet is a key factor shaping the gut microbiome (109). In turn, the gut microbiome also determines glycemic responses to certain foods, which show a high interpersonal variability based on individual microbiome features (110). Thus, it is imperative to unravel the basic metabolic processes and dynamic interactions of microorganisms that are linked to nutrition and diet. Since dietary SLs are essential components of eukaryotic cellular membranes, they can be found in virtually any type of food. Yet, our knowledge about the capability of microorganisms to degrade dietary SLs is limited. Just recently, first evidence on the microbial assimilation of dietary sphinganine in the mouse gut has been established by using a click-chemistry based approach (termed ClickSSS) to track the incorporation of bio-orthogonal dietary omega-alkynyl sphinganine into the gut microbial community (111). Bacteria from the *Bacteroides* genus were almost exclusively involved in the assimilation of sphinganine, although other non-SL-producing bacteria (e.g. *Bifidobacterium, Lactobacillus*, and *Turicibacter*) have been discerned to have a role in SL metabolism as well. In the future this and similar approaches should be used to identify yet uncharacterized microbial processes implicated in SL metabolism. Investigating the influence of dietary SLs on the gut microbiota is key to understand the tight connections between SL metabolism,the gut microbiota, and host immune homeostasis.

### SL-Mediated Host-Pathogen Interactions

**Figure 4:**
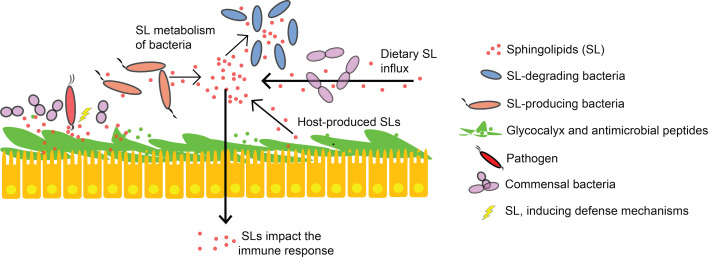
Sphingolipids in host-microbial interactions. Epithelial cells that produce various antimicrobial peptides and secrete mucin (glycocalyx) prevent microbial invasion. SLs alter intestinal mucus function, compete with commensals for epithelial binding sites and induce defense mechanisms against pathogenic bacteria. Intestinal SL levels are not only influenced by diet and endogenous SL-production, but also by SL-producing and SL-degrading bacteria. Bacterial and host SLs are structurally similar. They mediate specific host immune responses and interact with signaling pathways.

IECs and other intestinal cells are in permanent cross-talk with the microbiome and with bioactive compounds. It is supposed that SLs, e.g. SM, have protective effects at the mucosal site, thus being able to prevent the invasion of pathogenic microorganisms (20). SM is also taken up by enterocytes, which subsequently degrade the lipid backbone for reutilization (112). Degradation products of SLs and glycosphingolipids also interact with the immune system of the host (**Figure 4**). Sph and lyso-SLs, both derivatives from SL degradation, have antimicrobial properties against gram-positive and gram-negative pathogenic bacteria (e.g. *Pseudomonas aeruginosa, Staphylococcus aureus, Acinetobacter baumannii, Campylobacter jejuni, Listeria monocytogenes* and *Clostridium perfringens*), as shown in *in vitro* and *in vivo* experiments (20, 113, 114). Multiple epithelial tissues benefit from the preventive effect of Sph on severe pathogen infections involving innate and immediate defense mechanisms (113). The high expression of Sph in human nasal epithelial cells is associated with protective barrier effects, and decreasing Sph levels promote bacterial infections (115). Bacterial survival fluctuations during mouse lung infection were experimentally modified by deletion of a microbial sphingosine-responsive transcription factor (sphR), suggesting that sphR of pathogens plays an important role in the initial response to host infection (116). Also S1P has effects on infection dynamics *via* immune cell trafficking and differentiation as well as preserved barrier integrity (117, 118).

Glycosphingolipids act as antigens, as receptors for microbial products and toxins, and as mediators for cell adhesion in eukaryotic cells (119–121). They are suggested to have a functional role in host immune responses and for pathogen’s escaping strategies (122). Lactosylceramide (LacCer), a glycosphingolipid including Cer and lactose, has a special role in recognizing pathogen‐associated molecular patterns. It activates phagocyte function (123, 124) and there is evidence for direct binding to bacterial pathogens (shown for *Escherichia coli, Bordetella pertussis, Bacillus dysenteriae, Propionibacterium freudenreichii*) and fungi (shown for *Candida albicans*) (125–130). Increasing cellular Cer accumulation was found to parallel production of antimicrobial peptides (AMPs), which are key components of antimicrobial barrier functionality and innate immunity (131). C1P has been reported to directly activate cytosolic phospholipase A2 which subsequently leads to production of the AMPs human beta-defensin 2 and human beta-defensin 3 (132) Increased S1P levels were reported to strongly stimulate the expression of cathelicidin antimicrobial peptide when the epidermis is under stress, i.e. in response to attack of microbial pathogens (133). At the host neutrophils plasma membrane, LacCer accounts for 70% of glycosphingolipids, indicating its importance in pathogen binding (122, 134), and endogenous LacCer supplementation to neutrophil cell lines with low levels of LacCer can rescue their phagocytic activity (123).

## SL Homeostasis

The intense investigations on SLs in the last years revealed SL metabolism as a universal response to stress. Depending on the source and type of stress, different signaling pathways are activated, which provides explanations for the complex and often enigmatic nature of SLs. Recent innovations in systems biology, big data analysis, genomics and epigenetics enabled detailed analyses of SL structures resulting in highly interesting novel findings on the role of SLs in inflammation, disease progress and nutrition.

### SLs and the Drug Industry

The most intense studied bioactive SLs include Cer, Sph and S1P (135). The Sph analogue ISP1 (Myriocin) showed beneficial effects in treating insulin resistance and the metabolic syndrome (136, 137). Short-chain analogues of Cers are of special interest in the treatment of leukemia (138). Especially, S1P has been in the focus of many recent studies, as its two kinases (SphK 1 and 2) and its five G- protein coupled receptors (S1P1-5) received increased attention in research as therapeutic targets. Effects were shown in treatment approaches of diseases common for Western societies, for instance, ulcerative colitis (139), allergy (32), multiple sclerosis (140) and cancer (141). Furthermore, the SL metabolite is suspected to be involved in the onset and progression of psychiatric disorders and pain syndrome (75) which might provide evidence to find a missing link between GI inflammation and psychosomatic manifestations. The S1P analogue FTY720 (Fingolimod) was originally used in treatment of multiple sclerosis by modulating immune cell chemotaxis, but has been also suggested for cancer treatment (142) and for restoring endothelial barrier dysfunction (143).

Recent analyses focusing on the SL metabolism suggest C1P, cerebrosides (especially glucosylceramide and lactosylceramide) and gangliosides as novel emerging candidates for therapeutically targeting cancerogenic, immune-related and inflammatory disorders. Enzymes, especially SMases and ceramidases are in the center of recent investigations. Neutral SMase inhibitors show an enormous potential for treatment of inflammation in cardiovascular, pulmonary and neurological systems (144). Alkaline SMase is a promising target in GI disorders, due to its high activity in the digestive system. It has been suggested to regulate mucosal growth in an alkaline SMase knock-out mice model (145) and to reduce gut inflammation (93) in a rodent study. Acid SMase has been reported to reduce severity of cystic fibrosis (115), acute lung injury (146) and Wilson disease (147). When evaluating acid SMase as drug target, it is essential to mention, that the enzyme seems to work in a compartment specific manner. Thus, specific roles of acid SMases have to be further elucidated (148). Ceramidases have been suggested as promising treatment targets in many diseases. Inhibitors of acid ceramidase and neutral ceramidase overcome cell death resistance after prolonged anti-cancer treatments. Essential work is yet to be done to define inhibitors suitable for combining specificity and ability to reach specific cellular compartments (144). Although, promising candidates have been identified, their exact indication needs to be further investigated.

To date it remains unclear how to restore the SL balance in situations of altered SL homeostasis. However, there is strong evidence suggesting dietary SLs to be suitable candidates. Cers are well known targets when talking about skin barrier function. Reduced levels have been reported in situations of barrier dysfunction, as seen in psoriasis (149) and atopic dermatitis (150). Previously, the effect of supplementation of synthetic and animal-based Cer-precursor-SLs have been investigated. However, with the discovery of an SL efflux mechanism of rodent enterocytes (44) and the possibility to isolate the SLs from conventional plant-based food products, which diminished concerns about infectious diseases disseminating *via* animal SLs (151), plant SLs arespeculated to be safer. Beneficial effects of oral supplements have been indicated in studies on skin hydration and skin barrier reinforcement (56). A high abundance of gangliosides in human breast milk indicates them as beneficial dietary supplements in infant formula, due to their effects on GI inflammatory disorders and neurodevelopment of children (152–154). Altogether, there is increasing evidence supporting safety of dietary SL supplementation to counteract a dysregulated immune homeostasis. Nonetheless, information on differences regarding the source of the Cers (animal, plant, synthetic), structural differences of Cers and subsequent effects are not well studied to date.

### SL Metabolites - Novel Biomarkers for Stress Responses?

A dysfunction in SL homeostasis due to reoccurring stress stimuli might provide an explanation for many inflammatory and immune-related diseases of yet unknown origin. To support this theory SL metabolites have been suggested as novel biomarkers for diseases such as Alzheimer’s disease and metabolic diseases related to insulin resistance (155, 156). Cers have been identified as cholesterol-independent biomarkers for familial coronary artery disease by an unbiased machine learning approach, revealing 30 out of 32 Cer types being significantly elevated in sera of patients compared to healthy controls (157).

Establishing a biomarker system suitable for clinics depends on reliable measurements to distinguish health from disease. Several rodent and human studies found enhanced levels of SM, Cers, S1P and C1P associated with IBD and simultaneously reduced levels of glucosylceramides and monosialodihexosylgangliosides (77, 78, 158). A reduction of alkaline SMase activity was suggested to be a potential trigger for the dysregulation in SL metabolism as seen in a study of human chronic colitis (159). In a human clinical study, a high throughput whole exome sequencing approach was used to identify mutations in a chronic kidney disease and sensorineural hearing loss patient including also family members (160). This enabled the identification of a gene defect in RMND1, which leads to an accumulation of Cer and subsequently promote dysregulated apoptosis and tissue necrosis in kidneys. A broader accessibility to more advanced chemical analysis techniques such as UHPLC-High resolution mass spectrometry allows identification of SL species as biomarkers of clinical significance (161).Thus, not only methodological innovations will support sphingolipidomics, but also the dissemination of knowledge on accurate mass, isotopic patterns, and collision-induced fragmentation together with enlarged compound libraries suitable for identifying a broader spectrum of SL species is essential.

## Conclusion

SLs are important constituents of cell membranes and enable a fast and efficient transduction of cellular signals. Their highly complex metabolism is strictly regulated. Our diet has been demonstrated to influence SL homeostasis and to shape the gut microbial community. With regards to a balanced SL metabolisms, it is paramount to clarify the role of microbial SL producers and microbial SL metabolites. Western diets are hypothesized to dysregulate SL homeostasis and thereby cause an increase in prevalence of cancerogenic, immune-related and inflammatory diseases. In this context, immuno-nutrition can be a powerful tool to counteract chronic inflammation, overall morbidity, and increasing health care costs. Thus, it is imperative to further elucidate the SL metabolism and to define dietary recommendations in order to restore a dysregulated SL homeostasis. Furthermore, the impact of bacterial SLs on eukaryotic physiology, metabolic processes and immune homeostasis need to be in focus of future studies evaluating the role of SLs in immuno-nutrition. Recent technical innovations in system biology, genomics and epigenetics pave the way for such complex, holistic analysis of SLs. Additionally, unbiased machine learning approaches might constitute a key tool in upstream analysis of multidisciplinary data sets deriving from metabolomics, microbiome analysis and clinical studies. Although, many questions remain to be answered, it is clear that a detailed insight in the highly complex nature of SL homeostasis is pivotal to combat chronic, low-grade intestinal inflammation and subsequent metabolic diseases within the human body.

## Author Contributions

All authors listed have made a substantial, direct and intellectual contribution to the work, and approved it for publication.

## Funding

The research of the contributing authors on the topic of this review article has been funded by the Austria science fund FWF KLI284 and by grant 19056 of the Scientific funds of the major of the city of Vienna (both to EU). The research work of the authors on microbiome research is financially supported by the JPI HDHL KP project “Intestinal microbiomics” co-financed by the Austria Research Promotion Agency FFG (to ES and EU). The funders had no role in data collection, decision to publish, or preparation of the manuscript.

## Conflict of Interest

The authors declare that the research was conducted in the absence of any commercial or financial relationships that could be construed as a potential conflict of interest.
